# How does washing without water perform compared to the traditional bed bath: a systematic review

**DOI:** 10.1186/s12877-017-0425-4

**Published:** 2017-01-25

**Authors:** Fabian M. V. Groven, Sandra M. G. Zwakhalen, Gaby Odekerken-Schröder, Erik J. T. Joosten, Jan P. H. Hamers

**Affiliations:** 1 Department of Health Services Research, School for Public Health and Primary Care (CAPHRI), Maastricht University, Duboisdomein 30, 6229 GT Maastricht, The Netherlands; 2 Department of Health Services Research, School for Public Health and Primary Care (CAPHRI), Maastricht University, PO box 616, 6200 MD Maastricht, The Netherlands; 3 Department of Marketing & Supply Chain Management, School of Business and Economics, Maastricht University, Tongersestraat 53, 6211 LM Maastricht, The Netherlands; 4 Arion Holding, Vouersweg 103, 6161 AG Geleen, The Netherlands

**Keywords:** Bathing, Baths, Bed bath, Hygiene, Patient experiences, Review, Washing without water

## Abstract

**Background:**

For immobile patients, a body wash in bed is sometimes the only bathing option. Traditionally, the bed bath is performed with water and soap. However, alternatives are increasingly used in health care. Washing without water is one such alternative that has been claimed to offer several advantages, such as improved hygiene and skin condition. This systematic review aims to provide a comprehensive overview of the evidence on outcomes of the washing without water concept compared to the traditional bed bath.

**Methods:**

Controlled trials about washing without water outcomes published after 1994 were collected by means of a systematic literature search in CINAHL, Embase, MEDLINE, and PUBMED at the 25th of February, 2016. Additionally, references and citations were searched and experts contacted. Studies were eligible if (1) the study designs included outcomes of washing without water products developed for the full body wash compared to the traditional bed bath, and (2) they were controlled trials. Two researchers independently used a standardized quality checklist to assess the methodological quality of the eligible studies. Finally, outcomes were categorized in (1) physiological outcomes related to hygiene and skin condition, (2) stakeholder-related outcomes, and (3) organizational outcomes in the data synthesis.

**Results:**

Out of 33 potentially relevant articles subjected to full text screening, six studies met the eligibility criteria. Only two studies (of the same research group) were considered of high quality. The results of these high quality studies show that washing without water performed better than the traditional bed bath regarding skin abnormalities and bathing completeness. No differences between washing without water and the traditional bed bath were found for outcomes related to significant skin lesions, resistance during bathing and costs in the studies of high quality.

**Conclusions:**

There is limited moderate to high quality evidence that washing without water is not inferior to the traditional bed bath. Future research on washing without water is needed and should pay special attention to costs, hygiene, and to stakeholder-related outcomes, such as experiences and value perceptions of patients, nursing staff and family.

**Electronic supplementary material:**

The online version of this article (doi:10.1186/s12877-017-0425-4) contains supplementary material, which is available to authorized users.

## Background

A nurse enters Mr. Johnson’s room to give him a bed bath. Mr. Johnson is surprised that she only brings a small package with her instead of the washbasin with water, soap and towels that he would expect. After she explains to Mr. Johnson what she is going to do and after she uncovers him, the nurse opens the package and takes a washcloth. Mr. Johnson feels that the washcloth is wet when it is wiped over his face. After the first washcloth, seven other washcloths are used to bathe all his body parts separately while other body parts are covered again to mitigate cold and vulnerability. His skin dries within a minute, even though no towel is used. When the nurse is finished, she throws away all the washcloth, together with the empty package.

This hypothetical scenario represents the actual bathing practice in an increasing number of European and American health care institutions for immobile patients who, irrespective of the cause or level of immobility, need to be bathed in bed [1]. The package with washcloths in the example is often called “washing without water”, which can be described as disposable, prepacked products for the full body wash consisting of a non-woven carrier (washcloth) and a no-rinse cleansing fluid that allows nursing staff to bathe someone without the use of water [2]. This definition of washing without water does not include products for disinfection of the body or products used to sanitize certain body areas, such as the perineum.

Personal hygiene assistance, including bathing, is a key nursing activity that is reasoned to be related to quality of life and quality of care [3]. Bathing has been argued to have an effect on patient well-being [4] and patient comfort [5]. Furthermore, preserving skin integrity and personal hygiene are proposed to be important physiological functions of bathing, which in turn prevent infections and disease [6, 7].

The advantages of bathing in general also apply to the full body wash in bed (the bed bath), which is sometimes the only option to maintain hygiene for immobile patients who cannot bathe themselves due to chronic or acute illness. However, the most traditional bed bath with water, soap, towels and wash basins is argued to have some adverse outcomes. First of all, regarding stakeholder experiences, patients often feel dependent and uncomfortable during the traditional bed bath [8]. Therefore, the bed bath can trigger aggressive and agitated behaviors, especially if patients are cognitively impaired [3, 9, 10]. Consequently, the traditional bed bath can be burdensome for both patients and nursing staff [9, 10]. Furthermore, the physiological effectiveness of the traditional bed bath in terms of skin integrity and personal hygiene is questioned. For instance, Voegeli [11] found that both soap and towel drying disrupt the skin barrier function. In addition, bath basins [12, 13] and water [14] can contain pathogens related to hospital-acquired infections.

In 1990, the bag bath concept was developed, predominantly to tackle the shortcomings related to skin integrity and hygiene [15]. The bag bath concept consisted of a number of non-disposable washcloths put in a bag together with an amount of no-rinse cleansing fluid diluted in water. Washing without water evolved from the bag bath concept in 1994, and differs from this concept because it is an all-in-one (i.e. no separate water or cleansing fluid required) and completely disposable solution. Washing without water is claimed to offer several advantages compared to the traditional bed bath with water and soap. First of all, because water, soap, towels and wash basins are not needed, the negative effects on skin integrity and hygiene associated with these materials are eliminated [15, 16]. It is claimed that washing without water even has a positive influence on skin condition [17]. In addition, some authors claim that washing without water is less costly and less time-consuming than the traditional bed bath [16, 18, 19], which is relevant at an organizational level, for the management of health care institutions. Finally, washing without water is said to decrease physical and emotional strain and increase satisfaction among both patients and caregivers [20]. Some of these claimed outcomes are based on unpublished studies [17, 20]. It is unclear whether these are supported by scientific evidence.

Although patient hygiene is a core nursing responsibility, guiding evidence regarding skin cleansing practices, such as washing without water is missing [7, 21]. Such evidence could contribute to the adoption of effective skin cleansing practices in health care. At the moment, it is unclear whether washing without water is an effective skin cleansing practice and therefore a relevant alternative for bathing patients. Therefore, the objective of this systematic review is to assess the effectiveness of washing without water in comparison to the traditional bed bath for the full body wash of immobile patients.

## Methods

### Search strategy

A systematic literature search was executed in CINAHL, Embase, MEDLINE and PUBMED to collect controlled trials about the effectiveness of the washing without water concept. Studies published before 1995 were not included in the literature search, because only in that year the first article about the bag bath concept was published. Since the washing without water concept does not yet have a commonly accepted generic name, many combinations of adjectives and nouns were used that could represent the concept. Search terms were determined based on the key terms used in literature about washing without water to describe the concept. Identical terms as well as synonyms were used. Furthermore, no specific search terms regarding health care setting, type of patients or outcomes were included as we were interested in all effectiveness outcomes of washing without water for immobile patients in all settings. The search terms and filters used in the different databases are shown in Additional file 1. No language limiters were applied in any of the searches. The database search was executed at two points in time. The first search was performed in May, 2015, and in February, 2016, the search was updated. In the second search of February, 2016, only studies published after the date of the first search were retrieved from the databases. Both database searches consisted of two search runs. In the first search run, only studies labeled as a trial were obtained by using the trial filter. The second search run combined the search terms of the first search run with the search term ‘trial’ using the Boolean operator ‘AND’ and without using the trial filter to ensure that all trials were included in the search results. Additional studies were identified by screening references and by citation searching of the articles screened for full text. Finally, the list of studies was presented to experts in the field of washing without water to ensure that all relevant studies were included. These experts included an author of a previous study related to washing without water, and an infection prevention specialist who is knowledgeable about washing without water.

### Study selection

Studies were eligible if (1) the study designs included outcomes of washing without water products developed for the full body wash compared to the traditional bed bath, and (2) they were controlled trials. Conversely, studies about products specifically developed for other purposes than the regular body wash, such as incontinence care or disinfection, were excluded. Interventions that are related to washing without water but are excluded in this study are presented in Table 1, together with the reasons for exclusion. Finally, no criteria were determined regarding type of patients, nursing staff or health care settings to collect all evidence on outcomes of washing without water.

**Table 1:** Interventions which are related to washing without water but are excluded from this study

Intervention	Reason for exclusion
Barrier wipes that offer protection to the skin in the perineum	Although no water is used, these products are not used for the full body wash. Instead, these products offer protection to vulnerable skin.
Antibacterial wipes, such as CHG (Chlorhexidine Gluconate) wipes	Although no water is used, these products are not used for the full body wash but for killing bacteria on the skin and thereby disinfecting the skin. Some studies were found in which CHG wipes were compared to washing without water products. These studies did not include washing with water in the comparison or were not a controlled trial and therefore were excluded.
Original bag bath concept	When the original bag bath concept is used, non-disposable washcloths are put in a bag together with a no-rinse lotion diluted in water. Therefore, water still needs to be used to dilute the no-rinse solution. Furthermore, it is not a disposable solution.
No-rinse sprays and cleansing lotions	Although no water is used, these products are not prepacked so that one package includes the materials needed for the full body wash. Separate wipes are still needed and therefore it is not an all-in-one solution as described in our definition of washing without water.

### Study screening and data extraction

Studies were consecutively screened on title, abstract and full text for eligibility. To reduce bias, titles and abstracts were independently screened by two researchers (first author and MM) until the level of agreement between them exceeded 80%. From that moment, the remaining studies were screened by the first author alone. Studies were recorded as either “include”, “exclude” or “uncertain”, and disagreements between the researchers were discussed to reach consensus. Studies labelled as “include” or “uncertain” were included in subsequent stages of the screening process. Full text screening was done only by the first author. In case of doubt, the first author discussed the full text with one of the other authors (SZ), after which a collaborative decision was made to include or exclude the study.

Data were extracted from the relevant articles using a data extraction form specifically designed for the current review. This form included items needed for the quality assessment of the eligible studies such as items about the research design, study populations, type of interventions, study aims, statistical analyses, outcomes, and recommendations and limitations. For two articles, which lacked information regarding these items, the particular authors were contacted to retrieve additional data or the study protocol. Only one of these authors agreed to share the protocol.

### Methodological quality

Out of an inventory of several methodological quality assessment tools, the scale of Downs and Black [22] was selected to calculate a total quality score (Table 2). This scale is one of the most complete assessment tools and covers most risks of bias as described in the Cochrane Handbook for Systematic Reviews of Interventions [23]. Furthermore, the Agency of Healthcare Research and Quality (AHRQ) argues that the Downs and Black [22] scale is rigorously developed [24]. The original two items regarding blinding of persons receiving the intervention and of persons providing the intervention were not applied because this was considered to be practically impossible with the washing without water intervention. Furthermore, the scoring possibilities for the items related to the sample size and the description of principal confounders were adapted for this research (see Table 2). Two researchers (first author and HB) independently assessed study quality and discussed disagreements to reach consensus. When consensus was not reached, the particular items were discussed with one of the other authors (SZ). Based on the quality domains defined by the AHRQ, five items from the Downs and Black [22] scale were selected as representing the most important quality requirements. These items are related to the study aim description (item 1), statistical tests (item 16), randomization (item 21), intention-to-treat (item 23), and statistical power (item 25). In this review, a study was considered to be of high quality if it met all five of these items. If a study did not meet all of these criteria, it was considered to be of moderate quality.

**Table 2:** Quality assessment based on Downs and Black [22]

Items	Gillis et al. (2015) [26]	van Achterberg et al. (2015) [25]	Schoonhoven et al. (2015) [2]	Nøddeskou et al. (2015) [28]	Larson et al. (2004) [27]	Sheppard & Brenner (2000) [29]
Reporting						
1. Is the hypothesis/aim/objective of the study clearly described?^a^ *Yes = 1, No = 0*	1	1	1	1	1	1
2. Are the main outcomes to be measured clearly described in the introduction or methods section? *Yes = 1, No = 0*	1	1	1	1	1	1
3. Are the characteristics of the patients included in the study clearly described? *Yes = 1, No = 0*	1	1	1	1	1	1
4. Are the interventions of interest clearly described? *Yes = 1, No = 0*	1	1	1	1	1	1
5. Are the distributions of principal confounders in each group of subjects to be compared clearly described?^b^ *Yes = 1, No = 0*	1	1	1	0	0	0
6. Are the main findings of the study clearly described? *Yes = 1, No = 0*	1	1	1	0	1	1
7. Does the study provide estimates of the random variability in the data for the main outcomes? *Yes = 1, No = 0*	1	1	1	1	1	1
8. Have all important adverse events that may be a consequence of the intervention been reported? *Yes = 1, No = 0*	1	1	1	0	0	1
9. Have the characteristics of patients lost to follow-up been described? *Yes = 1, No = 0*	1	0	0	0	0	0
10. Have actual probability values been reported for the main outcomes except where the probability is less than 0.001? *Yes = 1, No = 0*	1	1	1	0	1	1
External validity						
11. Were the subjects asked to participate in the study representative of the entire population from which they were recruited? *Yes = 1, No = 0, Unable to determine = 0*	1	1	1	0	0	0
12. Were those subjects who were prepared to participate, representative of the entire population from which they were recruited? *Yes = 1, No = 0, Unable to determine = 0*	0	0	0	0	1	0
13. Were the staff, places, and facilities where the patients were treated representative of the treatment the majority of patients receive? *Yes = 1, No = 0, Unable to determine = 0*	1	1	1	1	1	0
Internal validity – bias						
14. If any of the results of the study were based on “data dredging”, was this made clear? *Yes = 1, No = 0, Unable to determine = 0*	0	1	1	1	0	0
15. Is the time period between the intervention and outcome the same for cases and controls? *Yes = 1, No = 0, Unable to determine = 0*	1	1	1	1	1	1
16. Were the statistical tests used to assess the main outcomes appropriate?^a^ *Yes = 1, No = 0, Unable to determine = 0*	1	1	1	0	0	0
17. Was compliance with the intervention reliable? *Yes = 1, No = 0, Unable to determine = 0*	0	1	1	1	1	0
18. Were the main outcome measures used accurate? *Yes = 1, No = 0, Unable to determine = 0*	1	1	1	0	0	0
Internal validity – confounding (selection bias)						
19. Were patients in different intervention groups or were the cases and controls recruited from the same population? *Yes = 1, No = 0, Unable to determine = 0*	1	1	1	1	1	1
20. Were the study subjects in different intervention groups or were the cases and controls recruited from the same population? *Yes = 1, No = 0, Unable to determine = 0*	1	0	0	1	1	1
21. Were study subjects randomized to intervention groups?^a^ *Yes = 1, No = 0, Unable to determine = 0*	1	1	1	1	0	0
22. Was the randomized intervention assignment concealed from both patients and health care staff until recruitment was complete and irrevocable? *Yes = 1, No = 0, Unable to determine = 0*	0	0	0	1	0	0
23. Was there adequate adjustment for confounding in the analyses from which the main findings were drawn?^a^ *Yes = 1, No = 0, Unable to determine = 0*	1	1	1	1	0	1
24. Were losses of patients to follow-up taken into account? *Yes = 1, No = 0, Unable to determine = 0*	1	1	1	0	0	0
Power						
25. Did the study have sufficient power to detect a clinically important effect where the probability value for a difference being due to chance is less than 5%?^ab^ *Yes = 1, No = 0*	0	1	1	0	0	0
Total quality score	20/25	21/25	21/25	14/25	13/25	12/25
Important quality items met	4/5	5/5	5/5	3/5	1/5	2/5
Excluded original items for this study: • Was an attempt made to blind study subjects to the intervention they have received? • Was an attempt made to blind those measuring the main outcomes of the intervention? Reason for exclusion: it is practically impossible to blind study subjects and those who measure outcomes for the washing without water intervention.						

^a^Important quality items that needed to be met to be considered as a study of high quality

^b^The original answer posibilities (5: Yes = 2, Partially = 1, No = 0 and 25: <n1 = 0, n1 - n2 = 1, n3 - n4 = 2, n5 - n6 = 3, n7 - n8 = 4, n8 + = 5) have been adapted

### Data synthesis and analysis

An overview of the evidence related to all washing without water outcomes was made. We categorized findings on the effectiveness of washing without water in (1) physiological outcomes related to hygiene and skin condition, (2) stakeholder-related outcomes, and (3) organizational outcomes. All results of the studies of moderate or high quality are presented.

## Results

### Search results

**Fig. 1:**
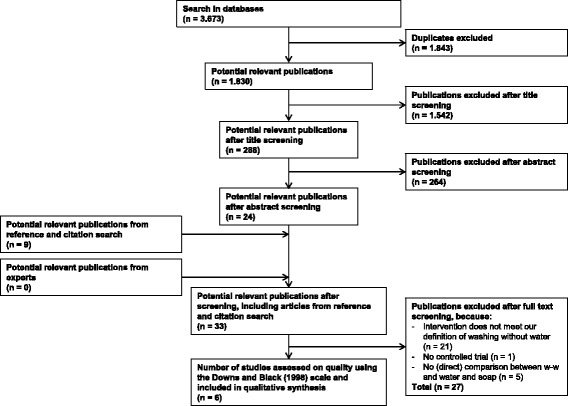
PRISMA flow diagram. Legend: A total of 6 studies were used to inform the findings for this manuscript. None of the studies labeled as “uncertain” based on title or abstract screening turned out to be eligible. Flow diagram adapted from Moher, Liberati, Tetzlaff, Altman, and THE PRISMA Group (2009) [37]

Figure 1 shows the results of the screening process. The numbers of studies mentioned in the figure are the totals of the articles retrieved in May, 2015, and February, 2016. Of the 1,830 unique studies that were retrieved from the electronic database search, ultimately, six studies were included in this review. The main reason for exclusion was that the intervention in the particular studies was not a washing without water product.

### Study characteristics and quality

Information regarding the characteristics of all included studies is provided in Table 3. The studies employed different study designs, including three randomized controlled trials [2, 25, 26]. The studies of Schoonhoven et al. and van Achterberg et al. were based on the same research data [2, 25]. Two other studies applied a controlled cross-over design over two consecutive days in which all subjects received the traditional bed bath on 1 day and a bed bath with a washing without water product on the other day [27, 28]. The order in which subjects received the different bed baths was randomized in only one of these studies [28]. The last study had a quasi-experimental design [29].

**Table 3:** Study characteristics

Author, year and country	Study design	Setting and sample population at baseline	Study duration
Gillis et al. (2015) [26] Belgium	Cluster controlled trial	Institutionalized long-term care 6 wards in 2 nursing homes 163 Residents with an average age of 84.9 years	12 weeks
van Achterberg et al. (2015) [25] The Netherlands	Cluster controlled trial	See Schoonhoven et al. (2015) [2] for setting and sample as the same dataset was used	6 weeks
Schoonhoven et al. (2015) [2] The Netherlands	Cluster controlled trial	Institutionalized long-term care 56 wards in 22 nursing homes 500 Residents: 61.6% Were diagnosed with dementia; average age of 82.4 years 275 Nurses	6 weeks
Nøddeskou et al. (2015) [28] Denmark	Randomized controlled cross- over trial	One hospital 65 Patients with an average age of 73 years 6 Nurses	2 days
Larson et al. (2004) [27] United States	Cross-over trial	Hospital – Three intensive care units of one hospital 47 Patients with an average age of 60.7 years 40 Nurses	2 days
Sheppard & Brenner (2000) [29] United States	Controlled time series trial	Institutionalized long-term care One nursing home 35 Residents: 56.25% Were cognitively impaired; average age of 85.17 years 11 Nurses	6 weeks

Moreover, research settings and populations also differed between the studies. Two studies [27, 28] were conducted in a hospital, and four [2, 25, 26, 29] took place in institutionalized long-term care. Furthermore, the four studies conducted in institutionalized long-term care only included residents aged 65 years or older (the average ages ranged from 82.4 to 85.17 years), whereas the two studies situated in hospitals also included patients younger than 65 years (leading to average ages of 73 [28] and 60.7 [27] years). Information regarding cognitive impairment as a feature of the study population was only reported in three studies [2, 25, 29], the details of which are mentioned in Table 3. The study by Nøddeskou et al. [28] excluded cognitively impaired patients, whereas the other two studies did not mention cognitive impairment as an exclusion criteria nor did they report on the presence of such patients [26, 27].

Information about the quality assessment of the studies can be found in Table 2. Only the studies by Schoonhoven et al. and van Achterberg et al. met all five of the primary quality items and were therefore considered to be of high quality [2, 25]. These studies also received the highest total quality scores of all included studies (21/25). The other four studies were considered to be of moderate quality. Of these four, the study of Gillis et al. [26] did obtain a high total score, but was not considered to be of high quality as it did not meet all of the five primary quality items (4/5). Due to the small number of studies, all studies of moderate to high quality were included in the data synthesis. Furthermore, because only a limited number of studies was found and the reported outcomes varied considerably between studies, it was not possible to assess the risk of bias across studies nor to conduct a meta-analysis.

Table 4 shows a complete overview of the results per included study. Skin hydration [26], any skin abnormalities, significant skin lesions [2], reduction in microbial counts [27], and skin dryness [29] were categorized as physiological outcomes. Resident satisfaction [2, 29], nurse satisfaction [2, 27–29], bathing completeness [25], and resistance during bathing [2] were regarded to be stakeholder-related outcomes. Finally, organizational outcomes were costs (including staff wages and material costs) [2, 27, 28], time of a bed bath [27, 28], and the quality score of the bed bath [27, 28].

**Table 4:** Results regarding washing without water outcomes

Study	Washing without water outcome	Sample population at end of study	Results
Gillis et al. (2015) [26]	Skin hydration (stratum corneum) at the leg, hand, and cheek	150 Residents in pre-test and post-test stages (108 in the intervention group and 42 in the control group)	The post minus pre skin hydration scores were higher for the intervention group (washing without water) compared to the control group (traditional bed bath) at the cheek (*p* = 0.02) showing a higher increase in skin hydration for this skin site in the intervention group.
van Achterberg et al. (2015) [25]	Bathing completeness	450 Residents (257 in the intervention group and 193 in the control group)	Bathing completeness was more often found in the intervention group (washing without water) (*p* < 0.0001). When all body parts were cleaned, bathing was considered complete.
Schoonhoven et al. (2015) [2]	Any skin abnormalities	450 Residents (257 in the intervention group and 193 in the control group)	There is a difference in prevalence of any skin abnormalities over time (*p* = 0.04). The number of skin abnormalities decreased in the intervention group (washing without water) and increased in the control group (traditional bed bath). Any skin abnormalities included bright red discoloration, erythema, white, green or yellow discoloration of the wound bed, atrophic and shiny skin, satellite lesions and kissing lesions, fissures, erosions, or ulcerations on the buttocks, eyes, neck, armpits, elbows, sub-mammary region, umbilicus, abdomen, groins, anal cleft, or the skin between the toes.
	Significant skin lesions	450 Residents (257 in the intervention group and 193 in the control group)	There is no difference in the prevalence of significant skin lesions over time between the intervention group and the control group (*p* = 0.82). If the skin on the buttocks, or in any of the skin folds in the sub-mammary region, abdomen, groins, or the anal cleft was not intact, the severity of the skin damage was considered to be significant.
	Nurse satisfaction	275 Nurses	Nurses gave an average grade of 7.5 (out of 10) for washing without water with a standard deviation of 1.2. 61% Of the nurses would replace water and soap bed baths with washing without water.
	Resident satisfaction	55 Residents	Residents gave an average grade of 7.1 (out of 10) for washing without water with a standard deviation of 2.0. 94% Thought washing without water cleaned the skin sufficient or good and 83% felt at least sufficiently fresh after being washed with washing without water. 61% Would permanently replace water and soap bed baths with washing without water.
	Resistance during bathing	450 Residents (257 in the intervention group and 193 in the control group)	There is no treatment by time interaction (*p* = 0.713). Resistance is observed by nurses and present if the resident: wards the nurse off, avoids the nurse’s touch, is restless, turns away, struggles with the nurse, or protests verbally.
	Costs	206 Observations of traditional baths and 272 observations of washing without water	There is no difference in costs at a confidence interval of 0.95. The total average costs over a time period of 6 weeks was €218,30 for washing without water and €232,20 for the traditional bed bath. The costs related to the nursing time needed to clean up after a bed bath were excluded from the calculation.
Nøddeskou et al. (2015) [28]	Nurse satisfaction	Preferences of 6 nurses for 54 individual bed baths	Nurses had a preference for washing without water compared to the traditional bed bath (*p* < 0.01).
	Costs	58 Observations of traditional baths and 58 observations of washing without water	The average total costs of a washing without water bed bath in Danish Krone was 106.25 (11.84 material costs and 94.41 costs related to the salary of the nurse) compared to 126.96 for the traditional bed bath (11.87 material costs and 115.09 costs related to the salary of the nurse). Costs related to the use of machinery and electricity were excluded from the calculation.
	Time of a bed bath	58 Observations of traditional baths and 58 observations of washing without water	Less time was used during all stages of the bed bath (preparation, the bath itself, and cleaning up) and in total when washing without water was used compared to the traditional bed bath (*p* < 0.001 for all).
	Quality score	58 Observations of traditional baths and 58 observations of washing without water	Eight quality factors of the bed bath were checked and rated on a Likert scale from 1 to 10. The quality factors included (1) gathering bathing equipment, (2) wearing gloves, (3) explaining procedure to patient, (4) checking patients’ well-being, (5) ensuring patients’ privacy, (6) avoiding recontamination of patients’ skin, (7) cleaning all body surfaces, and (8) disposing equipment without environmental contamination, The mean total quality score for washing without water was 8 (out of 10) compared to a mean quality score of 7.9 (out of 10) for the traditional bed bath.
Larson et al. (2004) [27]	Reduction in microbial counts from the umbilicus	29 Paired observations	There was no difference in the total bacterial counts between washing without water and the traditional bed bath (*p* = 0.47) after the bed bath, although the number of microbial counts increased in the washing without water group (*p* = 0.04).
	Reduction in microbial counts from the groin	33 Paired observations	There was no difference in the total bacterial counts between washing without water and the traditional bed bath after the bed bath (*p* = 0.78).
	Reduction in microbial counts of gram-negative bacteria from the groin	33 Paired observations	There was no difference in the total bacterial counts between washing without water and the traditional bed bath (*p* = 0.22) after the bed bath, although the number of gram-negative bacteria counts decreased in the traditional bed bath group (p = 0.04).
	Nurse satisfaction	40 Nurses	Nurses preferred washing without water over the traditional bed bath on the items related to convenience, time-consumption, patient comfort, required supplies, and overall preference (*p* < 0.001 for all). The only item for which no preference was expressed was about which bath type is more likely to clean and moisturize the skin (*p* = 0.20).
	Costs	44 Observations of traditional baths and 44 observations of washing without water	The total average costs were $18.15 for washing without water compared to $19.87 for the traditional bed bath. Costs related to the use of water, heating and sewage (in case of the traditional bed bath) were excluded from the calculation.
	Time of a bed bath	43 Observations of traditional baths and 43 observations of washing without water	The mean bath time was 12.8 min for washing without water compared to 14.4 min for the traditional bed bath. The total time did not differ between the two types of bed baths (*p* = 0.08). The time needed for the preparation of the bed bath and for cleaning up after the bed bath were excluded from the calculation. Instead, nurses were asked to estimate the time needed to prepare a bed bath. Nurses (*n* = 40) estimated that this would take significantly less time when a washing without water product is used.
	Quality score	43 Observations of traditional baths and 43 observations of washing without water	The total quality score was 5.88 for washing without water compared to 5.51 for the traditional bed bath (*p* = 0.25). The quality of the bed bath was assessed by checking eight quality items. The quality items included (1) gathering bathing equipment, (2) wearing gloves, (3) explaining procedure to patient, (4) checking patients’ comfort and safety, (5) ensuring patients’ privacy, (6) avoiding recontamination of patients’ skin, (7) cleaning all body surfaces, and (8) disposing equipment without environmental contamination, The bed bath received a score of one point for each item met, resulting in a maximum score of 8.
Sheppard and Brenner (2000) [29]	Skin dryness	30 Residents (16 in the intervention group and 14 in the control group)	The total skin condition differed between the two types of bed baths (*p* < 0.001). The total mean scores were stable in the control group (traditional bed bath) and improved over time in the intervention group (washing without water). More specifically, there was a difference between the groups in flaking (*p* < 0.001) and scaling (*p* = 0.001).
	Nurse satisfaction	11 Nurses	91% Of the nurses (strongly) agreed that washing without water was easy to administer and that residents were satisfied with this type of bed bath. 73% Thought washing without water was better for the skin of the resident compared to the traditional bed bath. 70% Thought the resident’s skin was clean after the bed bath with washing without water. 73% Indicated that washing without water was a worthy alternative for the traditional bed bath.
	Resident satisfaction	7 Residents of the intervention group (washing without water)	All (strongly) agreed that the product was easy to use and all liked the bathing technique. 86% Felt clean and indicated they had a softer skin after the bed bath with washing without water. 71% Indicated washing without water was a worthy alternative for the traditional bed bath.

### Results of physiological washing without water outcomes

Outcomes related to hygiene and skin condition are regarded to be physiological outcomes of washing without water in this review. The high quality study by Schoonhoven et al. [2] measured outcomes related to residents’ skin condition, being any skin abnormalities and significant skin lesions. If the skin of certain parts of the body was not intact, the severity of the skin lesion was considered to be significant, whereas skin abnormalities included, amongst others, erythema and skin discoloration. A significant difference in the prevalence of skin abnormalities over time (6 weeks) was observed between the washing without water group, in which the prevalence decreased, and the traditional bed bath group, in which the prevalence increased. However, no significant difference was found regarding the occurrence of significant skin lesions over time. Other findings related to the skin condition were presented in the studies of moderate quality from Sheppard and Brenner [29] and Gillis et al. [26]. The results of Sheppard and Brenner [29] showed a significant difference between the washing without water group and the traditional bed bath group regarding skin dryness in favor of washing without water. Furthermore, Gillis et al. [26] concluded that skin hydration at the cheek increased significantly more when washing without water was adopted compared to the traditional bed bath. No differences were found for skin hydration at hands or legs. Physiological outcomes related to hygiene were only reported in the study of moderate quality by Larson et al. [27], where no significant differences were found.

### Results of stakeholder-related washing without water outcomes

Next to physiological outcomes, stakeholder-related outcomes are considered to be important in this review. In a study of high quality, Schoonhoven et al. [2] reported a resident satisfaction score of 7.1 (out of 10) and a nursing staff satisfaction score of 7.5 for washing without water. However, they did not compare satisfaction regarding washing without water with the level of satisfaction regarding the traditional bed bath. Also Sheppard and Brenner [29] studied resident and nurse satisfaction without comparing the level of satisfaction between the two bathing methods in their study of moderate quality. A comparison of nursing staff satisfaction was made in the studies of moderate quality by Larson et al. [27] and Nøddeskou et al. [28]. Nursing staff in both studies significantly preferred washing without water over the traditional bed bath. The high quality study outcomes “bathing completeness” and “resistance during bathing” are also considered to be stakeholder-related outcomes in this review. Bathing completeness was found to be significantly higher for washing without water compared to the traditional bed bath [25]. Finally, no significant treatment by time interaction was found regarding resistance during bathing, which means that residents bathed with washing without water did not show more or less resistance compared to residents bathed the traditional way [2].

### Results of organizational washing without water outcomes

Finally, several findings related to organizational outcomes of washing without water compared to the traditional bed bath were reported. The only study of high quality that reported on costs did not find a significant difference in the total average costs over a time period of 6 weeks between washing without water and the traditional bed bath [2]. In two other studies of moderate quality, the average total costs of a bed bath were reported to be DKK 106.25 [28] and $18.15 [27] for washing without water and DKK 126.96 and $19.87 for the traditional bed bath, respectively. An important costs component is the nursing time spent on the bed bath. The complete time from preparation of the bed bath to clean-up was only studied in one study of moderate quality in which a significant difference was found both in the total bed bath time and in the time of all individual stages of the bed bath, which were all shorter for washing without water (*p* < 0.001) [28]. No significant time difference was found in the study of moderate quality of Larson et al. [27], who did not include the time needed for preparation and clean up. Finally, the quality of the bed bath was measured in two studies of moderate quality in which no difference between the two bathing methods was found [28, 29].

## Discussion

Evidence on outcomes of washing without water compared to the traditional bed bath is scarce. Six controlled trials on the effects of washing without water were found, of which only two were regarded to be of high quality [2, 25]. Moreover, these two studies were of the same research group and used the same dataset for the analysis. Nevertheless, the results of the few studies that have been conducted show that washing without water is not inferior to, and on some outcomes even outperforms the traditional bed bath. Washing without water performed significantly better than the traditional bed bath with respect to skin abnormalities [2], skin dryness/hydration [26, 29], nurse satisfaction [27, 28], and bathing completeness [25], of which only skin abnormalities and bathing completeness have been studied in studies of high quality. Furthermore, one study found a time difference [28], whereas the time of the bed bath did not differ in another study [27]. Both studies were assessed to be of moderate quality but only the former included the total time of a bed bath from preparation to clean-up. In addition, no significant differences between washing without water and the traditional bed bath were found for significant skin lesions, resistance during bathing or costs, in one of the studies of high quality [2]. Furthermore, studies of moderate quality did not find significant differences for microbial counts [27] or the quality of the bed bath [27, 28].

### Reflection on findings

Reflecting on the evidence on washing without water, we yield some noticeable findings. First, none of the studies included all cost components in the cost calculation. For example, Schoonhoven et al. [2] did not include the time that nursing staff spent cleaning up after the bed bath. Because nursing time is an important cost component related to the bed bath, substantial costs may not have been included in the cost calculation. Second, patient satisfaction between washing without water and the traditional bed bath has not been compared in any of the included studies, which is interesting given the growing attention for patient-centered care. The Institute of Medicine defines patient-centered care as care that suits patients’ needs and preferences [30]. Consequently, patient-centered care is argued to contribute to patient satisfaction [31]. The scarce evidence regarding the effect of washing without water on patient satisfaction could be explained by the output-oriented management approach in health care. From an output perspective, the bed bath is merely provided to reach cleanliness [32]. However, evidence on the effectiveness of washing without water regarding hygiene is also still scarce, as it is measured in only one of the included studies [27].

Finally, washing without water is increasingly used in health care institutions, while the evidence on washing without water in general, and on hygiene and patient satisfaction in particular, is limited. Thoma-Lürken et al. [1] also conclude that the effectiveness of many implemented health care interventions, such as washing without water, need to be further assessed. One could take the argument of Feo and Kitson [32], that bathing is often perceived as a low-priority nursing task, to explain the lack of evidence regarding washing without water [19, 33]. However, the number of studies about bathing and hygiene that we encountered while conducting this review demonstrates that the nursing field is highly interested in patient hygiene. Therefore, we encourage nurse researchers to actively cooperate with clinical researchers to conduct more trials of high quality that inform nursing staff on how to provide the best possible care, and consequently, to upgrade the perceived value of fundamental care [32].

### Research implications

The scarce evidence regarding washing without water in general, and regarding washing without water outcomes related to costs, hygiene and patient satisfaction in particular, calls for additional research. Although costs were measured in half of the included studies [2, 27, 28], not all costs were included in the measurements. A complete cost overview would contribute to the burden of proof regarding the cost-effectiveness of both bathing methods. Furthermore, the only study that reported on hygiene outcomes did not find a significant difference in total microbial counts from the groin or the umbilicus after the bed bath between washing without water and the traditional bed bath [27].

From a patient-centered care perspective, patient-related outcomes should be studied more elaborately, including patient satisfaction, patient experiences, and patients’ value perceptions regarding washing without water. According to Berwick [34], patient experiences lie at the heart of quality of care. The focus on patient experiences is consistent with patient-centered care and is likely to contribute to patient satisfaction [31]. Patient experiences are not only related to patient satisfaction and to quality of care, but also to the patient’s perceptions of the value of care [35]. For washing without water, this means that patients evaluate the value of the washing without water bed bath based on their experiences with it.

However, the patient is not the only stakeholder involved in the bed bath. The nursing staff is another important stakeholder group. Nursing staff may decide whether or not to use a washing without water product without providing the patient a choice. Moreover, family members are important stakeholders as they generally are highly involved in health care, especially when patients are old and vulnerable. Different stakeholders have diverse perceptions of value depending on their individual needs and contexts [36]. For example, patients might derive value from the experience of having soft skin, whereas the nursing staff might derive value from giving an emotionally and physically burden-free bed bath. Hence, not only should future research on washing without water consider bed bath experiences of patients, but it should also consider bed bath experiences of other stakeholders, such as nursing staff and family. Moreover, it should take into account stakeholders’ different value perceptions of washing without water.

Based on the importance of experiences and value perceptions of patients, nursing staff, and family members, another recommendation for future research is to study the relationship between the outcomes of washing without water which can be measured objectively, on the one hand, and stakeholders’ experiences and value perceptions on the other hand (e.g. by adopting a mixed method approach). For example, one of the outcomes of washing without water is that it is more time-effective compared to the traditional bed bath. We do not know whether this has a positive or a negative effect on the patients’ or nursing staff’s experiences of the bed bath. On the one hand, a shorter bed bath can be less burdensome and therefore positively affect experiences and value perceptions of patients and nursing staff. Moreover, time-effectiveness of washing without water might be positively related to bathing completeness as argued by van Achterberg et al. [25]. On the other hand, patients and nursing staff might perceive a shorter bed bath as being impersonal and therefore a reduction in bed bath quality [2]. By elaborately examining patients’ and nursing staff’s experiences, valuable information can be obtained about the importance of washing without water outcomes for the experience and the value perceptions of patients and nursing staff.

Finally, this review points to two methodological implications. First, future research is needed to come to credible generalizations about the effectiveness of washing without water. The two studies of high quality were conducted in an institutionalized long-term care setting. Therefore, especially for outcomes that are unique to these studies, such as bathing completeness [25] and skin abnormalities [2], it would be interesting to study the outcomes of these studies in other settings as well. Furthermore, while three of the included studies included patients with cognitive impairments [2, 25, 29], and one study excluded cognitively impaired patients [28], two studies did not report on the presence of such patients [26, 27]. Hence, it is unclear whether results of the studies that excluded cognitively impaired patients are generalizable to settings where these patients are present. The inclusion of cognitively impaired patients in half of the included studies demonstrates that washing without water might be particularly valuable for this patient population. Second, many different outcome measures have been used in the included studies and the internal validity is not always described. The use of outcome measures with high internal validity would enhance the reliability of guiding evidence related to bathing practices, such as washing without water.

### Limitations of this review

Two limitations of this study are related to the quality assessment of eligible studies adopted for this review. First of all, the Downs and Black [22] quality checklist does not provide a cut-off value that can be used to distinguish high quality studies from studies of lower quality. Hence, we selected five key criteria that needed to be met to be qualified as a study of high quality. The selection of other key criteria might have led to different quality assessment results. However, since all results of studies of moderate to high quality were included, we did not miss important outcomes regarding the effectiveness of washing without water. The second limitation regarding the quality assessment is that we did not assess the risk of reporting bias. We only asked authors of eligible studies for the study protocol in cases where information that we needed for the quality assessment was missing. We did not compare the study protocols of all included studies with the published results and therefore, were unable to assess whether authors did report on all intended outcomes. Moreover, the classification of outcomes as physiological, stakeholder-related or organizational is based on our own judgment. Although this classification is arbitrary, we did not miss important outcomes as all reported outcomes are included in one of the outcome groups.

To our best knowledge, the current study is the first systematic review of washing without water. This review provides a complete overview of the evidence on washing without water as we included all controlled trials irrespective of the outcomes studied, type of patients or health care setting. Our eligibility criteria are based on a restricted definition of washing without water, excluding bathing concepts and products such as the bag bath concept and antibacterial wipes. Furthermore, studies that did not adopt a controlled trial design were excluded from this review. Although some may consider that our chosen criteria limit the search results, we consider the criteria to be a strength of this study. By excluding concepts such as the bag bath, we assured that the results were related to the exact same intervention, being washing without water. In our opinion, the inclusion of related but distinct concepts would have clouded the results. Moreover, we argue that the inclusion of non-controlled trials would have weakened the results.

## Conclusions

Although only a few studies related to washing without water in comparison to the traditional bed bath were found, this review offers valuable evidence to health care institutions by indicating that washing without water can be seen as a worthy alternative to the traditional bed bath. Because the two studies of high quality were conducted in an institutionalized long-term care setting, the evidence is particularly relevant to long-term care institutions. The results show that washing without water does not underperform compared to the traditional bed bath. Additionally, washing without water performs better on some outcomes. Consequently, compared to the traditional bed bath, washing without water might offer more advantages and value to the patient and the nursing staff, but possibly also to other stakeholders such as family members and the management of health care institutions. Especially because the washing without water concept is increasingly used in health care, there is a need for additional research to substantiate the advantages of washing without water. Future research should particularly focus on hygiene outcomes, and on patients’, nursing staff’s and family members’ value perceptions and experiences related to washing without water.

## Additional file

Additional file 1: Search strategies used in PUBMED, MEDLINE, CINAHL and Embase. Search strategies used in different databases. (PDF 164 kb)

## Abbreviations

AHRQ: Agency of Healthcare Research and Quality
CHG: Chlorhexidine Gluconate
