# Supplementary material for: How does washing without water perform compared to the traditional bed bath: a systematic review
Source: BMC Geriatr. 2017 Jan 25;17:31. doi: 10.1186/s12877-017-0425-4 (PMC5264342; doi:10.1186/s12877-017-0425-4)
Supplement: Additional file 1: — Search strategies used in PUBMED, MEDLINE, CINAHL and Embase. Search strategies used in different databases. (PDF 164 kb) [file 12877_2017_425_MOESM1_ESM.pdf]

Additional file 1: Search strategies used in PUBMED, MEDLINE, CINAHL and Embase

Search strategy PUBMED

Search run 1 as performed at the 25th of February, 2016

(Prepack* OR pack* OR disposable* OR "no rinse" OR “non rinse” OR impregnated OR wet OR "single use" OR care OR "Without water" OR waterless OR basin OR basins OR washbasin* OR washbowl OR alternative OR alternatives OR towel OR bag OR "one step" OR dry)

(in title/abstract)

AND

(wipe OR wipes OR cloth OR cloths OR washcloth* OR glove OR gloves OR mitt OR mitts OR cleanser* OR wash OR washing OR bath OR bathing OR "bed bath" OR "bed wash" OR "body wash")

(in title/abstract)

OR

(Bath MeSH term)

(in all fields)

Filters used:

- Articles published between 1995 – current
- “Clinical trial” or “controlled trial” or “randomized controlled trial”

Search run 2 as performed at the 25th of February, 2016

(Prepack* OR pack* OR disposable* OR "no rinse" OR “non rinse” OR impregnated OR wet OR "single use" OR care OR "Without water" OR waterless OR basin OR basins OR washbasin* OR washbowl OR alternative OR alternatives OR towel OR bag OR "one step" OR dry)

(in title/abstract)

AND

(wipe OR wipes OR cloth OR cloths OR washcloth* OR glove OR gloves OR mitt OR mitts OR cleanser* OR wash OR washing OR bath OR bathing OR "bed bath" OR "bed wash" OR "body wash")

(in title/abstract)

OR

(Bath MeSH term)

(in all fields)

AND

(trial)

(in all fields)

Filters used:

- Articles published between 1995 – current

Search strategy MEDLINE

Search run 1 as performed at the 25th of February, 2016

(Prepack$4.ab,ti. OR pack$4.ab,ti. OR disposable*.ab,ti. OR "no rinse".ab,ti. OR "non rinse".ab,ti. OR impregnated.ab,ti. OR wet.ab,ti. OR "single use".ab,ti. OR care.ab,ti. OR "Without water".ab,ti. OR waterless.ab,ti. OR basin.ab,ti. OR basins.ab,ti. OR washbasin$1.ab,ti. OR washbowl.ab,ti. OR alternative.ab,ti. OR alternatives.ab,ti. OR towel.ab,ti. OR bag.ab,ti. OR "one step".ab,ti. OR dry.ab,ti.)

AND

(wipe.ab,ti. OR wipes.ab,ti. OR cloth.ab,ti. OR cloths.ab,ti. OR washcloth*.ab,ti. OR glove.ab,ti. OR gloves.ab,ti. OR mitt.ab,ti. OR mitts.ab,ti. OR cleanser*.ab,ti. OR wash.ab,ti. OR washing.ab,ti. OR bath.ab,ti. OR bathing.ab,ti. OR "bed bath".ab,ti. OR "bed wash".ab,ti. OR "body wash".ab,ti.)

OR

(Sub Heading Bath)

(in all fields; “Instrumentation”, “Nursing”, “Methods” and “Standards” were selected)

Filters used:

- Articles published between 1995 – current
- “Clinical trial” or “controlled trial” or “randomized controlled trial”

Search run 2 as performed at the 25th of February, 2016

(Prepack$4.ab,ti. OR pack$4.ab,ti. OR disposable*.ab,ti. OR "no rinse".ab,ti. OR "non rinse".ab,ti. OR impregnated.ab,ti. OR wet.ab,ti. OR "single use".ab,ti. OR care.ab,ti. OR "Without water".ab,ti. OR waterless.ab,ti. OR basin.ab,ti. OR basins.ab,ti. OR washbasin$1.ab,ti. OR washbowl.ab,ti. OR alternative.ab,ti. OR alternatives.ab,ti. OR towel.ab,ti. OR bag.ab,ti. OR "one step".ab,ti. OR dry.ab,ti.)

AND

(wipe.ab,ti. OR wipes.ab,ti. OR cloth.ab,ti. OR cloths.ab,ti. OR washcloth*.ab,ti. OR glove.ab,ti. OR gloves.ab,ti. OR mitt.ab,ti. OR mitts.ab,ti. OR cleanser*.ab,ti. OR wash.ab,ti. OR washing.ab,ti. OR bath.ab,ti. OR bathing.ab,ti. OR "bed bath".ab,ti. OR "bed wash".ab,ti. OR "body wash".ab,ti.)

OR

(Sub Heading Bath)

(in all fields; “Instrumentation”, “Nursing”, “Methods” and “Standards” were selected)

AND

(trial.af.)

Filters used:

- Articles published between 1995 – current

Search strategy CINAHL

Search run 1 as performed at the 25th of February, 2016

(Prepack* OR pack* OR disposable* OR "no rinse" OR “non rinse” OR impregnated OR wet OR "single use" OR care OR "Without water" OR waterless OR basin OR basins OR washbasin* OR washbowl OR alternative OR alternatives OR towel OR bag OR "one step" OR dry)

(in abstract)

AND

(wipe OR wipes OR cloth OR cloths OR washcloth* OR glove OR gloves OR mitt OR mitts OR cleanser* OR wash OR washing OR bath OR bathing OR "bed bath" OR "bed wash" OR "body wash")

(in abstract)

OR

(Bath MeSH term)

(in all fields; “Bathing” and “Baths” were selected)

Filters used:

- Articles published between 1995 – current
- “Clinical trial” or “randomized controlled trial”
- Apply related words

Search run 2 as performed at the 25th of February, 2016

(Prepack* OR pack* OR disposable* OR "no rinse" OR “non rinse” OR impregnated OR wet OR "single use" OR care OR "Without water" OR waterless OR basin OR basins OR washbasin* OR washbowl OR alternative OR alternatives OR towel OR bag OR "one step" OR dry)

(in abstract)

AND

(wipe OR wipes OR cloth OR cloths OR washcloth* OR glove OR gloves OR mitt OR mitts OR cleanser* OR wash OR washing OR bath OR bathing OR "bed bath" OR "bed wash" OR "body wash")

(in abstract)

OR

(Sub Heading Bath)

(all fields; “Instrumentation”, “Nursing”, “Methods” and “Standards” were selected)

AND

(trial)

(in all fields)

Filters used:

- Articles published between 1995 – current
- Apply related words

Search strategy Embase

Search run 1 as performed at the 25th of February, 2016

(Prepack$4.ab,ti. OR pack$4.ab,ti. OR disposable*.ab,ti. OR "no rinse".ab,ti. OR "non rinse".ab,ti. OR impregnated.ab,ti. OR wet.ab,ti. OR "single use".ab,ti. OR care.ab,ti. OR "Without water".ab,ti. OR waterless.ab,ti. OR basin.ab,ti. OR basins.ab,ti. OR basinless.ab,ti. OR washbasin$1.ab,ti. OR washbowl.ab,ti. OR alternative.ab,ti. OR alternatives.ab,ti. OR towel.ab,ti. OR bag.ab,ti. OR "one step".ab,ti. OR dry.ab,ti.)

AND

(wipe.ab,ti. OR wipes.ab,ti. OR cloth.ab,ti. OR cloths.ab,ti. OR washcloth*.ab,ti. OR glove.ab,ti. OR gloves.ab,ti. OR mitt.ab,ti. OR mitts.ab,ti. OR cleanser*.ab,ti. OR wash.ab,ti. OR washing.ab,ti. OR bath.ab,ti. OR bathing.ab,ti. OR "bed bath".ab,ti. OR "bed wash".ab,ti. OR "body wash".ab,ti.)

Filters used:

- Articles published between 1995 – current
- All clinical trials

Search run 2 as performed at the 25th of February, 2016

(Prepack$4.ab,ti. OR pack$4.ab,ti. OR disposable*.ab,ti. OR "no rinse".ab,ti. OR "non rinse".ab,ti. OR impregnated.ab,ti. OR wet.ab,ti. OR "single use".ab,ti. OR care.ab,ti. OR "Without water".ab,ti. OR waterless.ab,ti. OR basin.ab,ti. OR basins.ab,ti. OR basinless.ab,ti. OR washbasin$1.ab,ti. OR washbowl.ab,ti. OR alternative.ab,ti. OR alternatives.ab,ti. OR towel.ab,ti. OR bag.ab,ti. OR "one step".ab,ti. OR dry.ab,ti.)

AND

(wipe.ab,ti. OR wipes.ab,ti. OR cloth.ab,ti. OR cloths.ab,ti. OR washcloth*.ab,ti. OR glove.ab,ti. OR gloves.ab,ti. OR mitt.ab,ti. OR mitts.ab,ti. OR cleanser*.ab,ti. OR wash.ab,ti. OR washing.ab,ti. OR bath.ab,ti. OR bathing.ab,ti. OR "bed bath".ab,ti. OR "bed wash".ab,ti. OR "body wash".ab,ti.)

AND

(trial.af.)

Filters used:

- Articles published between 1995 – current
